# Protein serine/threonine phosphatase PPEF-1 suppresses genotoxic stress response via dephosphorylation of PDCD5

**DOI:** 10.1038/srep39222

**Published:** 2017-01-04

**Authors:** Soo-Yeon Park, Jaesung Seo, Hyo-Kyoung Choi, Hye-Jeong Oh, Garam Guk, Yoo-Hyun Lee, Jeongmin Lee, Woo Jin Jun, Kyung-Chul Choi, Ho-Geun Yoon

**Affiliations:** 1 Department of Biochemistry and Molecular Biology, Brain Korea 21 PLUS Project for Medical Sciences, Yonsei University College of Medicine, Seoul, South Korea; 2 Division of Nutrition and Metabolism Research Group, Korea Food Research Institute, Gyeonggi-do, South Korea; 3 Department of Food and Nutrition, The University of Suwon, Kyunggi-do 445-743, South Korea; 4 Department of Medical Nutrition, Kyung Hee University, Kyunggi-do, 446-701, South Korea; 5 Department of Food and Nutrition, Chonnam National University, Gwangju, South Korea; 6 Department of Biomedical Sciences, and Department of Pharmacology, University of Ulsan College of Medicine, Seoul, South Korea

## Abstract

Programmed cell death 5 (PDCD5) is believed to play a crucial role in p53 activation; however, the underlying mechanism of how PDCD5 function is regulated during apoptosis remains obscure. Here, we report that the serine/threonine phosphatase PPEF-1 interacts with and dephosphorylates PDCD5 at Ser-119, which leads to PDCD5 destabilization. Overexpression of wild-type PPEF-1, but not inactive PPEF-1^D172N^, efficiently suppressed CK2α-mediated stabilization of PDCD5 and p53-mediated apoptosis in response to etoposide (ET). Conversely, PPEF-1 knockdown further enhanced genotoxic stress responses. Notably, PPEF-1 suppressed p53-mediated genotoxic stress response via negative regulation of PDCD5. We also determined that overexpression of wild-type PPEF-1, but not inactive PPEF-1^D172N^, significantly increased tumorigenic growth and chemoresistance of A549 human lung carcinoma cells. Collectively, these data demonstrate that PPEF-1 plays a pivotal role in tumorigenesis of lung cancer cells by reducing PDCD5-mediated genotoxic stress responses.

Coordinated regulation of cell death is crucial for the maintenance of cellular homeostasis^1^. Aberrant regulation of cell death is believed to lead to the development of multiple diseases such as neurodegeneration, immune deficiency, infertility, and cancer^2^. It is well known that p53 is a key executioner of cellular apoptosis regulation^3^. In unstressed conditions, the p53 protein is degraded by MDM2. In response to cellular stress, such as DNA damage, oxidative stress, and hypoxia, p53 rapidly accumulates in the nucleus and undergoes posttranslational modifications^4^, then becomes activated by interacting proteins such as ASPPs, Brn-3b, NF-kB/p52, and Muc1, leading to an increase in cellular apoptosis^5^,^6^,^7^,^8^. Programmed cell death 5 (PDCD5) associates with p53 in response to DNA damage and stabilizes p53 by inhibiting the interaction between MDM2 and p53^9^. PDCD5 also binds to and activates the histone acetyltransferase Tip60, which promotes DNA damage responses^10^. PDCD5 also participates in the release of cytochrome C by mediating the translocation of cytosolic Bax to the mitochondria^11^. PDCD5 downregulation has been reported in multiple human cancers due to its anti-apoptotic activity^2^,^12^,^13^,^14^. Despite the crucial role of PDCD5 in p53 activation and apoptosis, the factors and mechanisms involved in modulating PDCD5 function have not been elucidated. Casein kinase 2 was recently shown to phosphorylate PDCD5 at Ser-199 *in vitro*^10^. That study showed that overexpression of the phosphor-PDCD5 mutant, which replaced Ser-119 with alanine, failed to increase p53 activity, suggesting that CK2 positively affected PDCD5 function via Ser-119 phosphorylation. More recently, our group reported that CK2-dependent phosphorylation enhanced the stabilization and nuclear localization of PDCD5, which leads to p53-mediated genotoxic stress responses^15^. However, it is unclear how PDCD5 phosphorylation is regulated during genotoxic stress responses.

Accumulating evidence indicates that reversible protein phosphorylation is executed by kinases and phosphatases^16^. For example, the PDCD5-interacting protein p53 is phosphorylated at Ser-15 by ATM and this phosphorylation is removed by p53-induced phosphatase 1 (Wip1), which consequently prevents p53-dependent G2 arrest^17^. DUSP26 inhibits doxorubicin-induced p53 phosphorylation at Ser-20 and Ser-37, and consequently inhibits p53-mediated downstream activity^18^. Similar to p53, PDCD5 rapidly accumulates in the nucleus and is associated with the p53 response to genotoxic stress^19^. A recent study suggests that the phosphorylation status of PDCD5 affects it pro-apoptotic function^10^. In agreement with these reports, we found that CK2 knockdown diminished the levels of PDCD5 and phosphorylated PDCD5, and reduced p53 activation in response to etoposide (ET)^15^. This raises the possibility that PDCD5 function could be negatively regulated by dephosphorylation; however, the phosphatase that regulates PDCD5 phosphorylation has not been identified.

Here, we report that the protein serine/threonine phosphatase PPEF-1 binds to PDCD5 and reduces its phosphorylation at Ser-119, which consequently inhibits p53-mediated apoptosis. We show that PPEF-1 overexpression significantly enhances tumor growth and chemoresistance of A549 cells. Our results identify the molecular mechanism by which PPEF-1 negatively regulates the pro-apoptotic function of PDCD5 via PDCD5 dephosphorylation.

## Results

### Protein serine/threonine phosphatase PPEF-1 binds to and dephosphorylates PDCD5 at Ser-119

**Figure 1:**
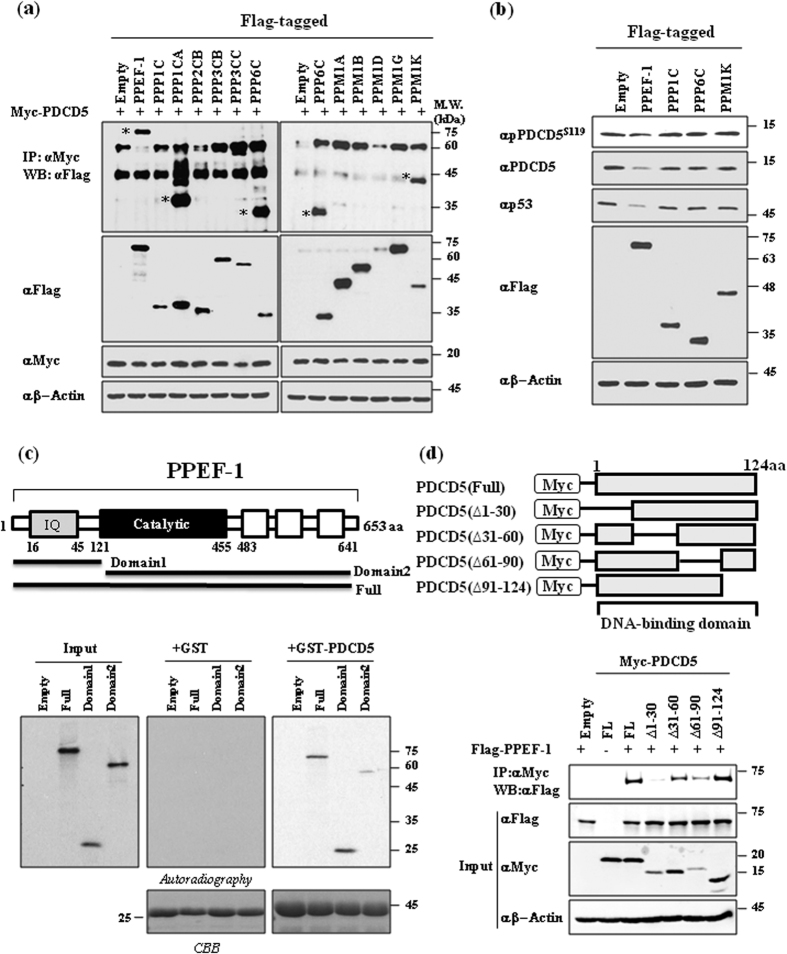
The protein serine/threonine phosphatase PPEF-1 binds to and dephosphorylates Ser-119 of PDCD5. **(a)** PDCD5 interacts with PPEF-1, PPP1CA, PPP6C, and PPM1K. Myc-tagged PDCD5 and Flag-tagged phosphatases were co-transfected into HCT116 (*p53*^+/+^) cells. Whole-cell lysates were immunoprecipitated and immunoblotted with the indicated antibodies. **(b)** PPEF-1 selectively dephosphorylates PDCD5 at Ser-119. Cell lysates were immunoblotted with the indicated antibodies. **(c)** The IQ motif domain of PPEF-1 interacts with PDCD5. Schematic diagrams of Myc-tagged PPEF-1 for GST pull-down analysis. Bound proteins were eluted and analyzed using autoradiography. **(d)** The N-terminal domain of PDCD5 (residues 1–30) interacts with PPEF-1. Schematic diagrams of Myc-tagged PDCD5 for co-immunoprecipitation and mapping analysis. HCT116 (*p53*^+/+^) cells were cotransfected with the indicated Myc-PDCD5 and Flag-PPEF-1 plasmids. Cell lysates were immunoprecipitated with Flag antibodies, and subsequently immunoblotted with the indicated antibodies. Full-length blots are presented in Supplementary Figure 1a-d.

We recently showed that PDCD5 phosphorylation at Ser-119 is rapidly induced by the genotoxic stress response, which stabilizes the protein and results in PDCD5 nuclear translocation^15^. PDCD5 phosphorylation is maintained at a low level in the absence of genotoxic stress. It is believed that protein phosphorylation is reversibly regulated by kinases and phosphatases^16^. Therefore, we examined whether a specific phosphatase dephosphorylates PDCD5 at Ser-119. First, we screened for the phosphatase that interacts with PDCD5. We generated a library of mammalian expression vectors that encode 12 serine/threonine phosphatases, including 6 protein phosphatases (PPPs), 5 metal-dependent protein phosphatases (PPMs) and protein phosphatase with EF-hand domain 1(PPEF-1). We co-transfected PDCD5 and various phosphatases into the human colorectal carcinoma cell line HCT116, and analyzed the associations of PDCD5 with these phosphatases by immunoprecipitation using antibody against Myc. Four phosphatases, including PPEF-1, PPP1CA, PPP6C, and PPPM1K, strongly interacted with Myc-PDCD5 (Fig. 1a). PPEF-1 overexpression specifically reduced PDCD5 phosphorylation at Ser-119 and PDCD5 protein level. Consequently, PPEF-1 also reduced the level of p53 (Fig. 1b). GST pull-down analysis showed that PPEF-1 directly interacted with PDCD5. Mapping analysis demonstrated that the domain 1 region, including the IQ domain of PPEF-1 (residues 1–30), directly interacted with PDCD5 (Fig. 1c). Next, we examined the PPEF-1-binding region in PDCD5. Co-immunoprecipitation analysis showed that the PDCD5 N-terminal domain (residues 1–30) interacted with PPEF-1 (Fig. 1d). These data suggest that PPEF-1binds to and dephosphorylates PDCD5 at Ser-119 during the genotoxic stress response.

### PPEF-1 suppresses etoposide-induced PDCD5 phosphorylation and stabilization

**Figure 2:**
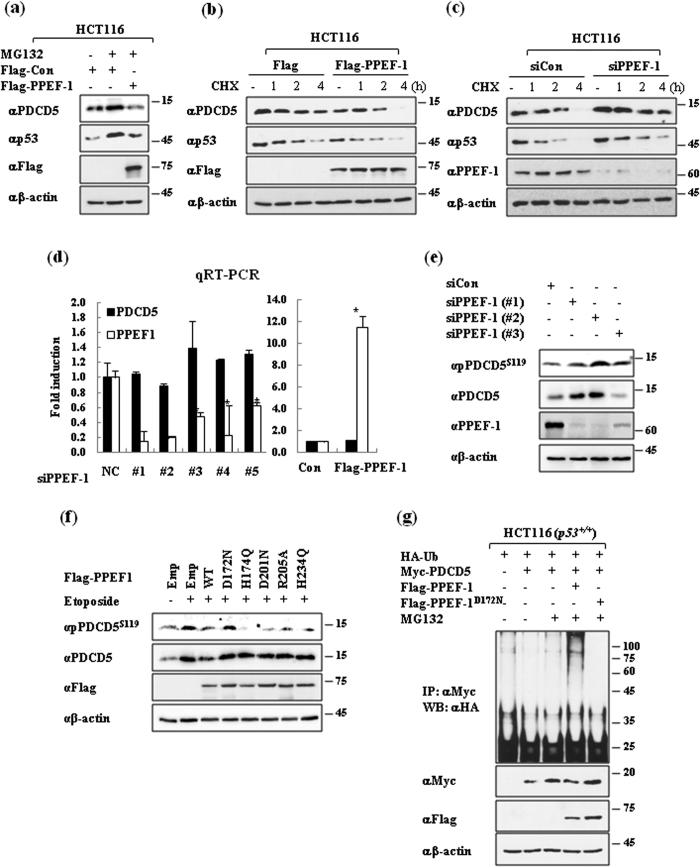
PPEF-1 dephosphorylates PDCD5, which causes protein destabilization via the ubiquitin-dependent proteasomal degradation pathway. (**a**) PPEF-1 overexpression blocks the effect of MG132 on PDCD5 stabilization. HCT116 cells were transfected with Flag-tagged PPEF-1. Two days after transfection, cells were treated with MG132 (10 μM) for 6 h. Total lysates were immunoblotted with the indicated antibodies. (**b**,**c**) PPEF-1 reduces the stability of PDCD5 protein. HCT116 cells were transfected with either Flag-PPEF-1 or si-PPEF1, cells were treated with 10 μg/mL cycloheximide (CHX) for the indicated times, and then immunoblotted with the indicated antibodies. (**d**) PPEF-1 does not affect the mRNA level of PDCD5. A549 cells were transfected with either Flag-PPEF-1 or si-PPEF1, and qRT-PCR analyses were performed to measure PDCD5 mRNA expression levels. *P < 0.05 versus control. (**e**) PPEF-1 knockdown increases PDCD5 phosphorylation. Cells were harvested and immunoblotted 48 h after siRNA transfection. (**f**) Inactive PPEF-1^D172N^ mutant does not affect PDCD5 Ser-119 phosphorylation. Forty-eight hours after transfection, cells were treated with 50 μM etoposide (ET) for 2 h and then harvested. Cell lysates were immunoblotted with the indicated antibodies. (**g**) PPEF-1 overexpression increases PDCD5 ubiquitination. HCT116 cells were transfected with HA-tagged ubiquitin, Flag-tagged PPEF-1, Flag-tagged PPEF-1^D172N^, or Myc-tagged PDCD5 as indicated. Cells were treated with MG132 (10 μM) for 6 h before harvesting. Total cell extracts were immunoprecipitated using the Myc antibody and immunoblotted with the HA antibody. Full-length blots are presented in Supplementary Figure 2a-g.

We found that PPEF-1 directly bound to and dephosphorylated PDCD5. Next, we investigated whether PPEF-1 reduced PDCD5 stabilization via the ubiquitin-dependent proteosomal pathway. Treatment with MG132 significantly increased PDCD5 levels, whereas PPEF-1 overexpression diminished the effect of MG132 on PDCD5 stabilization (Fig. 2a). A cycloheximide time-course experiment showed that PPEF-1 overexpression further reduced PDCD5 stability when compared with that of the control (Fig. 2b). We next examined the effect of PPEF-1 knockdown on PDCD5 stability using siRNAs designed for PPEF-1. The specific knockdown efficiency of each siRNA was established by western blotting and quantitative RT-PCR analysis (Fig. 2c–e). Then, we examined the change in PDCD5 mRNA levels in A549 cells after treatment with the siRNAs for PPEF-1. PPEF-1 knockdown reduced PPEF-1 mRNA levels, but did not reduce PDCD5 mRNA levels, and PPEF-1 overexpression did not affect PDCD5 mRNA levels (Fig. 2d). Consistently, PPEF-1 knockdown significantly increased PDCD5 stability when compared with the effect of cycloheximide treatment alone, which indicates a crucial role for PPEF-1 in PDCD5 destabilization (Fig. 2c) PPEF-1 knockdown enhanced the phosphorylation and stabilization of endogenous PDCD5 (Fig. 2e). These results demonstrate that PPEF-1 reduces PDCD5 stability via PDCD5 dephosphorylation.

Next, we investigated whether PPEF-1 phosphatase activity is required for the reduction of PDCD5 phosphorylation and stabilization by generating PPEF-1 mutants in which the catalytic domain residues are replaced with alternative amino acid residues; we, then, tested for phosphatase activity. As expected, overexpression of wild-type PPEF-1 efficiently reduced the phosphorylation and stabilization of PDCD5. However, overexpression of the inactive mutant PPEF-1^D172N^ had negligible effects on the phosphorylation and stability of PDCD5 when compared with that of other PDCD5 mutants (Fig. 2f). We recently reported that the stability of PDCD5 is regulated via the ubiquitin-dependent proteasomal degradation pathway^15^. We examined the effect of PPEF-1 overexpression on changes in PDCD5 ubiquitination. Wild-type PPEF-1 overexpression dramatically increased PDCD5 ubiquitination; however, overexpression of inactive PPEF-1^D172N^ had a negligible effect on PDCD5 ubiquitination and stability (Fig. 2g). These combined results suggest that PPEF-1 dephosphorylates PDCD5, which leads to protein degradation via the ubiquitin-dependent proteasomal degradation pathway.

### PPEF-1 antagonizes CK2-mediated PDCD5 stabilization in response to genotoxic stress

**Figure 3:**
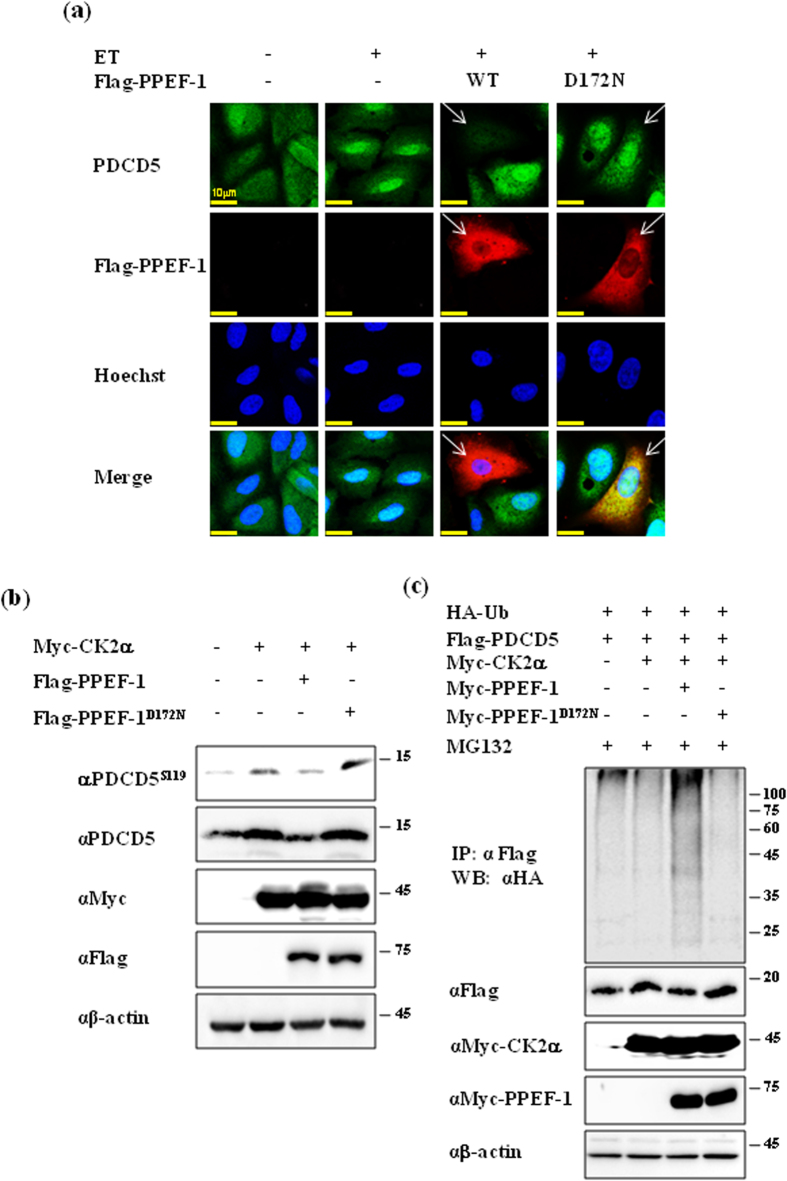
PPEF-1 antagonizes CK2 action on PDCD5 stabilization. **(a)** PPEF-1 overexpression induced PDCD5 degradation, but the inactive PPEF-1^D172N^ mutant did not affect ET-induced stabilization. A549 cells were transfected with the indicated constructs, and then treated with ET for 6 h. Fixed cells were stained with the indicated immunofluorescent antibodies. Bar scale = 10 μm **(b)** Active PPEF-1, but not the inactive PPEF-1^D172N^ mutant, antagonizes CK2-induced PDCD5 stabilization. A549 cells were transfected with Myc-CK2α or Flag-PPEF-1 as indicated. Total cell extracts were immunoblotted with the indicated antibodies. **(c)** Inactive PPEF-1^D172N^, but not wild-type PPEF-1, fails to antagonize CK2-induced reduction of PDCD5 ubiquitination. HCT116 cells were transfected with HA-tagged ubiquitin, Myc-tagged CK2, PPEF-1, PPEF-1^D172N^, or Flag-tagged PDCD5 as indicated. Cells were treated with MG132 (10 μM) for 6 h before harvesting. Total cell extracts were immunoprecipitated using Myc antibody and immunoblotted with HA antibody. Full-length blots are presented in Supplementary Figure 3b-c.

CK2-dependent phosphorylation enhanced PDCD5 stability and nuclear localization in response to genotoxic stress^15^. ET treatment rapidly induced nuclear accumulation of PDCD5, which was consistent with the result of a previous study^10^. Wild-type PPEF-1 completely abrogated PDCD5 stabilization in response to ET. However, the inactive PPEF-1^D172N^ mutant failed to block accumulation of PDCD5 in the nucleus, indicating that PPEF-1 has a crucial role in negative regulation of PDCD5 function (Fig. 3a).

We previously reported that CK2 phosphorylated PDCD5 at Ser-119, and thereby positively regulated its function. Next, we examined whether PPEF-1 antagonized CK2 activity and affected PDCD5 stability and phosphorylation. CK2 overexpression significantly increased PDCD5 phosphorylation and stability; however, overexpression of wild-type PPEF-1 efficiently reversed CK2-mediated PDCD5 phosphorylation and stability. By contrast, the inactive PPEF-1^D172N^ mutant failed to antagonize CK2-mediated PDCD5 phosphorylation and stability (Fig. 3b). Overexpression of wild-type PPEF-1, but not mutant PPEF-1^D172N^, dramatically increased PDCD5 ubiquitination (Fig. 3c). These combined results indicate that PPEF-1 antagonizes CK2 action on PDCD5 stabilization.

### PPEF-1 inhibits p53-mediated genotoxic stress response via negative regulation of PDCD5

**Figure 4:**
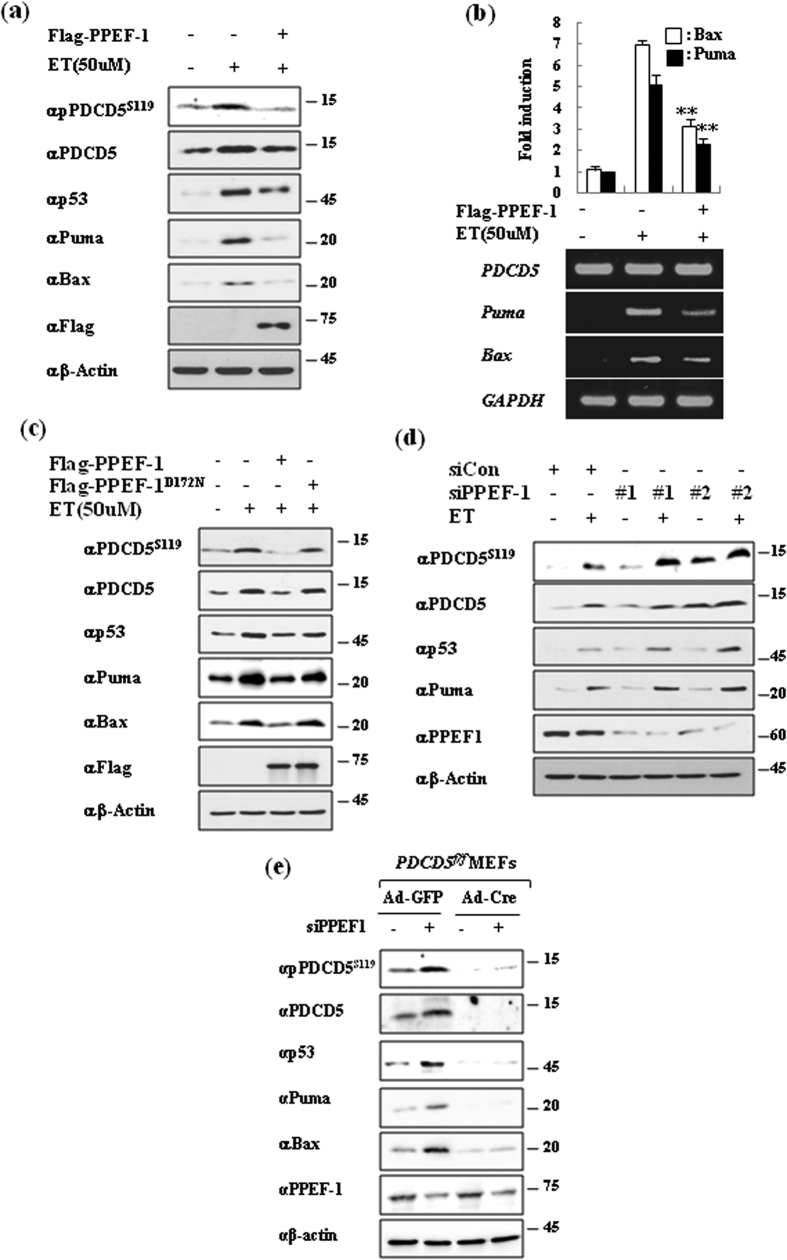
PPEF-1 inhibits p53-mediated genotoxic stress response via negative regulation of PDCD5. **(a)** PPEF-1 inhibits ET-induced p53 activation by PDCD5 dephosphorylation. A549 cells were transfected with Flag-tagged PPEF-1 and treated with ET. Cell lysates were immunoblotted with the indicated antibodies. **(b)** PPEF-1 reduces ET-induced p53 target gene expression. Puma and Bax mRNA expression were measured by qRT-PCR. **(c)** PPEF-1 phosphatase activity was necessary to inhibit ET-induced PDCD5 phosphorylation. Cells were transfected with Flag-tagged PPEF-1^WT^ or inactive PPEF-1^D172N^ mutant, and treated with ET for 6 h. Cell lysates were immunoblotted with the indicated antibodies. **(d)** PPEF-1 knockdown enhanced PDCD5-p53 signaling. A549 cells were transfected with siPPEF-1, and cell lysates were immunoblotted with the indicated antibodies. **(e)** PPEF-1 suppresses p53 activation via negative regulation of PDCD5. PDCD5^f/f^ MEF cells were infected with cre-Adenovirus for 24 h, and then transfected with siPPEF-1 for 48 h. Immunoblotting was performed with the indicated antibodies. Full-length blots are presented in Supplementary Figure 4a-e.

PDCD5 was identified as a positive regulator of p53-mediated apoptosis. Therefore, we examined the role of PPEF-1 on p53-mediated apoptosis signaling. PPEF-1 overexpression efficiently blocked p53 activation and transcriptional activation of pro-apoptotic genes in response to ET (Fig. 4a,b). However, the inactive PPEF-1^D172N^ mutant did not affect ET-induced p53 activation (Fig. 4c), and PPEF-1 knockdown significantly enhanced p53 activation in response to ET. These results indicate that PPEF-1 is a negative regulator of p53-mediated apoptosis (Fig. 4d).

We previously showed that PDCD5 is required for p53-mediated genotoxic stress response signaling. Therefore, we examined whether PPEF-1 inhibited p53 activation via PDCD5. Consistently, PPEF-1 knockdown substantially increased PDCD5 stability, phosphorylation, and p53 activation in mouse embryonic fibroblasts (MEFs). However, depletion of PDCD5 by treating *PDCD5*^*flox/flox*^ MEFs with Cre recombinase (Ad-Cre) expressing adenovirus blocked the effect of PPEF-1 knockdown on p53 activation (Fig. 4e). These results indicate that PPEF-1 suppresses p53 activation via negative regulation of PDCD5.

### Overexpression of PPEF-1 confers chemoresistance in human A549 lung cancer cells

**Figure 5:**
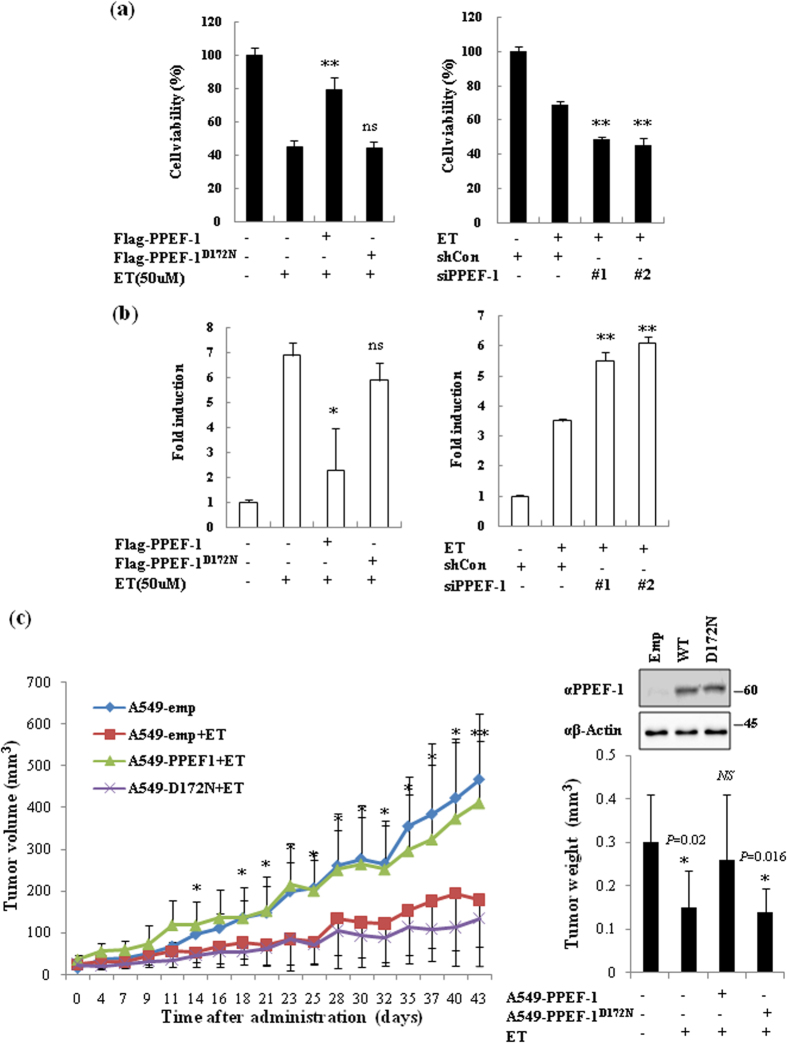
PPEF-1 overexpression confers chemoresistance in A549 human lung cancer cells. **(a)** PPEF-1 overexpression confers resistance to ET-induced cell apoptosis. A549 cells were transfected with either Flag-PPEF-1 or siRNAs. Cells were treated with ET after 24 h, and cell viability was determined using MTT assays. Error bars indicate standard deviation (SD; *n* = 3). **P < 0.01 versus control. **(b)** PPEF-1 knockdown increases ET-induced apoptosis in A549 cells. A549 cells were transfected with either Flag-PPEF-1 or siRNAs. Cells were treated with ET after 24 h and cell apoptosis was measured using TUNEL assays. Error bars indicate standard deviation (SD; *n* = 3). *P < 0.05, **P < 0.01 versus control. **(c)** PPEF-1 overexpression enhances the chemoresistance of A549 cells. PPEF-1 or PPEF-1^D172N^ stable overexpression cells were injected subcutaneously into the right flank of nude mice and allowed to grow for 2 weeks. Then, mice with comparable-sized tumors (~50 mm^3^) were selected for treatment with ET (10 mg/kg) at 2-day intervals for 6 weeks. Tumor volumes were measured every other day. *P < 0.05, **P < 0.01 versus A549-emp+ET. Error bars indicate SD (*n* = 6 per group). Full-length blots are presented in Supplementary Figure 5c.

To investigate the functional role of PPEF-1 in genotoxic stress-induced apoptosis, we performed MTT and TUNEL assays in A549 lung cancer cells after overexpression of wild-type PPEF-1 or inactive PPEF-1^D172N^ mutant. ET treatment increased DNA damage and cancer cell death, whereas PPEF-1 overexpression dramatically suppressed the ET-induced DNA damage response and cell death. However, the inactive PPEF-11^D172N^ mutant failed to suppress the DNA damage response and cancer cell death (Fig. 5a,b, left panel). PPEF-1 knockdown further increased the ET-induced apoptosis and cell death, demonstrating the negative regulatory function of PPEF-1 on cellular apoptosis (Fig. 5a,b, right panel). Given the critical role of PPEF-1 in blocking cell death in A549 cells, we next examined whether PPEF-1 overexpression increased *in vivo* chemoresistance of A549 cells. To this end, we generated stable A549 cell lines expressing wild-type PPEF-1 or mutant PPEF-1^D172N^ and performed xenograft assays using subcutaneous injections of the stable A549 cell lines into nude mice. The results showed that overexpression of wild-type PPEF-1 significantly increased the tumorigenic growth and chemoresistance of A549 cells compared with that of control cells. Strikingly, overexpression of the inactive PPEF-1^D172N^ mutant had only a negligible effect on chemoresistance of A549 cells (Fig. 5c). These results indicate that PPEF-1 overexpression enhances the chemoresistance of A549 lung cancer cells by reducing the genotoxic stress response.

## Discussion

PDCD5 is a positive regulator of p53 during the DNA damage response, and CK2 increases the levels of PDCD5 phosphorylation and stability. CK2-mediated phosphorylation of PDCD5 at Ser-119 is responsible for PDCD5 nuclear translocation in response to genotoxic stress, which correlates with p53 activation. These combined results indicate that CK2 positively enhances p53-mediated apoptosis via phosphorylation of PDCD5 at Ser-119. PDCD5 protein is maintained at a low level in the absence of genotoxic stress response, similar to p53; therefore, the dephosphorylation status of PDCD5 is likely mediated by a phosphatase that destabilizes PDCD5.

PPEF-1 function has only been partially described as involved in apoptosis in human cells. PPEF-1 was initially found to participate in the differentiation of sensory neurons, and was highly similar to *Drosophila* retinal degeneration C (RdgC)^20^. Loss-of-function studies of the *RdgC* gene increased light-dependent photoreceptor apoptosis in *Drosophila*^21^. This result is consistent with our finding that PPEF-1 suppresses apoptosis in response to genotoxic stress. PPEF-1 possesses three EF-hand motifs at the C-terminus, suggesting a role of PPEF-1 in calcium regulatory responses^22^. Calcium ion is an important second messenger that controls cell death responses. Calcium release from the endoplasmic reticulum to the cytosol is a requisite for apoptosis^23^. Changes in the cytosolic calcium level are associated with T-cell lymphoma^24^. *PPEF-1* transcript is strongly expressed in the T-cell lymphoblastic lymphoma cell line, suggesting a plausible role in the development of T-cell lymphoblastic lymphoma^25^. Therefore, it would be interesting to determine whether PPEF-1 modulates calcium-induced apoptosis via negative regulation of PDCD5 in T-cell lymphoblastic lymphoma cell.

Here, we identified four phosphatases, PPEF-1, PPP1CA, PPP6C, and PPM1K as PDCD5 interacting molecules among 12 phosphatases, and we found that only PPEF-1 dephosphorylates PDCD5 at Ser-119, which leads to protein destabilization via the ubiquitin-dependent proteosomal degradation pathway. PPEF-1 efficiently suppresses the p53-mediated genotoxic stress response. Notably, we did not observe the anti-p53 action of PPEF-1 in the depletion of PDCD5. This suggests that PPEF-1 suppresses p53 activation via dephosphorylation of PDCD5 Ser-119. It is known that phosphatases can function as oncogenes or tumor suppressors. Phosphatases can modulate a variety of signaling pathways, and dysregulation results in abnormal processes including uncontrolled proliferation, differentiation, angiogenesis, and metastasis. Therefore, many phosphatases have been associated with the development and progression of different types of cancer^26^. A549 lung cancer cell line was used as a model for cell signaling data and xenograft to demonstrate the PDCD5-p53 signaling pathway efficiently because A549 and HCT116 cell lines are suitable cell line to highlight PPEF1-PDCD5-p53 pathway. We also used HCT116 colorectal cancer cell line not only for immunoprecipitation, but for validating PPEF-1 mediated destabilization of PDCD5 since A549 and HCT116 cells were well established cell lines to see PDCD5-p53 signaling pathway. For reference, we found elevated level of PPEF-1 in colorectal, breast, prostate, liver, and lung cancer from THE HUMAN PROTEIN ATLAS (www.proteinatlas.org) website. Our data from the *in vivo* xenograft assay indicate that PPEF-1 overexpression increased the tumorigenic growth and chemoresistance of A549 cells, suggesting that PPEF-1 can act as an oncogene in lung cancer development by preventing cancer cell death. Further elucidation of PPEF-1 function in human cancer tumorigenesis will facilitate the development of targeted therapeutics for cancer treatment.

## Methods

### Cell culture and reagents

Human lung adenocarcinoma cell line A549 and human colorectal carcinoma cell line HCT116 were purchased from and authenticated by the Korean Cell Line Bank (Seoul, Korea) using short tandem repeat analysis. A549 cells were cultured in RPMI, and HCT116 and PDCD5^f/f^ MEF cells were cultured in Dulbecco’s modified Eagle’s medium (DMEM) supplemented with 10% (v/v) fetal bovine serum (FBS), 100 units/mL penicillin, and 0.1 mg/mL streptomycin (Hyclone, Logan, UT, USA) at 37 °C under 5% CO_2_. Etoposide, MG132, and cycloheximide (CHX) were purchased from Sigma-Aldrich (St. Louis, MO, USA) and prepared in dimethyl sulfoxide (DMSO) (Sigma-Aldrich). Lipofectamine 2000 reagent was used for transfection of overexpression constructs, and Lipofectamine RNAiMax reagent was used for siRNA transfection (Life Technologies, Grand Island, NY, USA).

### Plasmids and small interfering RNAs (siRNAs)

PPEF-1 and PDCD5 constructs were generated by PCR and cloned into the pSG5-KF2M1-FLAG, -Myc, or –HA vectors (Sigma-Aldrich), and the pGEX4T-1 vector (GE Healthcare, Piscataway, NJ, USA). All plasmid constructs were verified by DNA sequencing. For siRNA transfection, cells were plated at 60–70% confluency and transfected using Lipofectamine RNAiMax (Life Technologies) with 10 pM siRNA following the manufacturer’s protocol. The medium was changed after 4 h, cells were incubated for 2 days, and then treated with the indicated reagents. The following siRNA sequences were used: for siPPEF-1 #1, sense 5′-CACAUAUGAUUUAGAUAGAUU-3′ and antisense 5′-UCUAUCUAAAUCAUAUGUGUU-3′; for siPPEF-1 #2, sense 5′-GCAUUAGUACCUACAUAUUUU-3′ and antisense 5′-AAUAUGUAGGUACUAAUGCUU-3′; for siPPEF-1 #3, sense 5′-GAAGCAUUGACUUUAAUGAUU-3′ and antisense 5′-UCAUUAAAGUCAAUGCUUCUU-3′; for negative control, sense 5′-CCUCGUGCCGUUCCAUCAGGUAGUU-3′ and antisense 5′-CUACCUGAUGGAACGGCACGAGGUU-3′.

### Site-directed mutagenesis

Various mutants were created using the QuickChange kit (Agilent Technologies, Santa Clara, CA, USA). Site-directed mutagenesis was performed with 18 PCR amplification cycles under the following reaction conditions: denaturation at 94 °C for 30 s, annealing at 55 °C for 1 min, and extension at 68 °C for 10 min. Amplified mixtures were treated with *Dpn*I (Agilent Technologies) at 37 °C for 1 h, and aliquots were used to transform competent *E. coli*. All constructs were confirmed by DNA sequencing.

### Western blotting, immunoprecipitation, and antibodies

Cells were washed with cold phosphate-buffered saline (PBS) and collected. Cell extracts were prepared with lysis buffer (50 mmol/L Tris-Cl pH 7.5, 150 mmol/L NaCl, 1% NP40, 10 mmol/L NaF, 10 mmol/L sodium pyrophosphate, and protease inhibitors) and incubated on ice for 30 min. The lysates were centrifuged at 20,000 × *g* for 10 min at 4 °C. Total cell lysate protein was incubated with anti-Myc (Cell Signaling) or anti-Flag (Sigma) antibodies and 20 μL of protein A/G agarose overnight at 4 °C. Then, the agarose beads were washed with buffer three times, and the immunoprecipitated protein-antibody complexes were separated on SDS-PAGE gel and then transferred to nitrocellulose membranes. The membranes were blocked by incubating for 2 h in 5% (w/v) non-fat DifcoTM skim milk blocking buffer in 1× PBS with Tween-20 (PBST). The blocked membranes were incubated overnight at 4 °C with the indicated antibodies. The membranes were then washed with 1× PBST, incubated with the appropriate secondary anti-rabbit or anti-mouse horseradish peroxidase-conjugated antibody (Thermo Scientific, Rockford, IL, USA) for 1 h, and visualized using the LAS-3000 system (Fujifilm, Stamford, CT, USA) with an enhanced chemiluminescence detection reagent (Thermo Scientific). The following antibodies were used: anti-phospho PDCD5^S119^ (generated by AbFrontier, Seoul, South Korea), anti-PDCD5 (Proteintech, Chicago, IL, USA), anti-PPEF-1 and anti-HA (Santa Cruz Biotechnology, Dallas, Texas, USA), anti-Flag and anti-β-actin (Sigma-Aldrich), and anti-Myc (Cell Signaling, Danvers, MA, USA).

### RNA isolation and quantitative RT-PCR

Total RNA was extracted using the TRIzol reagent following the standard protocol (TAKARA, Shimogyo-ku, Kyoto, Japan). Then, cDNA was prepared using random hexamer primers (Chromogen, Seoul, Korea). PCR was performed using the following forward and reverse primers: human GAPDH, 5′-GATGGCATGGACTGTGGTCA-3′ and 5′-GCAATGCCTCCTGCACCACC-3′; human PPEF-1, 5′-GGCCAAATGCAGTTATCCAC-3′ and 5′-TCTACTTTGCCAGCAAACTGGTGC-3′; human PDCD5, 5′-AAAGCACAGGGAAGCAGAAA-3′ and 5′-TTGTCCATATCTTGCCATCTG-3′; human BAX, 5′-TCTACTTTGCCAGCAAACTGGTGC-3′ and 5′-TGTCCAGCCCATGATGGTTCTGAT-3′; 5′-GTGTTCCTACCCCCAATGTGT-3′. The cDNA concentration was normalized using GAPDH. Quantitative-PCR analyses were performed using SYBR Green PCR master mix reagents and an ABI Prism 7700 sequence detection system (Applied Biosystems, Carlsbad, CA, USA). All reactions were performed in triplicate. Relative expression levels and SD values were calculated using the comparative method.

### *In vivo* ubiquitination assay

HCT116 cells were co-transfected with plasmids encoding HA-Ub, Myc-PDCD5, Myc-CK2α, or Flag-PPEF1. After 48 h, whole-cell lysates were treated with MG132 for 6 h, and subsequently processed for immunoprecipitation with anti-Myc antibody. Ubiquitination of Myc-PDCD5 was visualized by western blotting with an anti-HA antibody.

### Immunofluorescence staining

Cells were cultured on coverslips, fixed in 4% paraformaldehyde for 30 min at 4 °C, and then treated with 0.4% Triton X-100 in PBS for 10 min at room temperature. Then, the fixed and permeabilized cells were incubated with anti-PDCD5 (Proteintech), anti-Myc (Cell Signaling), or anti-Flag (Sigma) antibodies at 37 °C for 2 h, and then stained with goat anti-rabbit FITC or goat anti-mouse rhodamine (Invitrogen) at 37 °C for 1.5 h. The nucleus was revealed with Hoechst 33258 staining. Cells were imaged using a Zeiss LSM700 confocal microscope (Carl Zeiss, Oberkochen, Germany).

### MTT assay

Cell viability was determined with the conventional MTT reduction assay. First, 5 × 10^3^ to 1 × 10^4^ cells were seeded in a 96-well plate and incubated overnight. Then, cells were transfected with the indicated plasmids for 30 h, and then treated with EGF or gefitinib for another 48 h. Cells were then treated with 15 μM MTT solution (Sigma-Aldrich) for 90 min at 37 °C. Formazan formation was resolved with DMSO solution for 30 min with shaking. The absorbance was recorded at 570 nm, and a reference was recorded at 630 nm with a microplate reader (Model 550, Bio-Rad Laboratories, Hercules, CA, USA).

### TUNEL assay

To detect cell apoptosis, DNA fragmentation was evaluated by performing a TUNEL assay using the HT Titer TACS Assay Kit (Trevigen, Gaithersburg, MD, USA) according to the manufacturer’s instructions. Briefly, the cells were fixed with 3.7% buffered formaldehyde solution for 7 min, washed with PBS, permeabilized with 100% methanol for 20 min, washed with PBS twice, digested with proteinase K for 15 min, quenched with 3% hydrogen peroxide, washed with distilled water, labeled with deoxynucleotidyl transferase, incubated at 37 °C for 90 min, and then treated with stop buffer. The cells were incubated with TACS-Sapphire substrate, and the colorimetric reaction was stopped with 0.2 N HCl after 30 min. The colorimetric reaction was measured in a microplate reader at absorbance 450 nm.

### Xenograft experiments

A suspension of 10^6^ A549 cells in 100 μL RPMI with Matrigel matrix (BD Science, Lexington, KY, USA) was injected subcutaneously into the left flank of 5-week-old male athymic BALB/c nu/nu mice (Orient, Seoul, Korea). Each experimental group included eight mice. Tumor size was monitored closely and measured every 2 days using a caliper. Two weeks after injection, mice with comparable-sized tumors (~50 mm^3^) were selected for treatment with ET (10 mg/kg) at 2-day intervals for 5 weeks. After 6 weeks of ET treatment, mice were sacrificed, and tumors were harvested, photographed, and weighed. Tumor volume was estimated using the formula: Volume = ½ × *a* × *b*^2^, where *a* and *b* represent the largest and smallest tumor diameters, respectively. Animal studies were performed in accordance with the National Institutes of Health guidelines and ethics guidelines of Yonsei University, and all animal procedures were approved by the Committee on Animal Investigations of Yonsei University and complied with the animal welfare act. The methods applied in this study were performed in accordance with the approved guidelines.

### Statistical analysis

Statistical significance was examined using Student’s *t*-test. The two-tailed *t*-test was used for comparisons of two groups. Values were reported as mean ± standard deviation (SD). *P* values < 0.05 were considered significant.

## Additional Information

**How to cite this article**: Park, S.-Y. *et al*. Protein serine/threonine phosphatase PPEF-1 suppresses genotoxic stress response via dephosphorylation of PDCD5. *Sci. Rep.* **7**, 39222; doi: 10.1038/srep39222 (2017).

**Publisher's note:** Springer Nature remains neutral with regard to jurisdictional claims in published maps and institutional affiliations.

## Supplementary Material

Supplementary Information
