# Supplementary material for: Protein serine/threonine phosphatase PPEF-1 suppresses genotoxic stress response via dephosphorylation of PDCD5
Source: Sci Rep. 2017 Jan 4;7:39222. doi: 10.1038/srep39222 (PMC5209732; doi:10.1038/srep39222)

Protein serine/threonine phosphatase PPEF-1 suppresses genotoxic stress response via dephosphorylation of PDCD5

Soo-Yeon Park[1,7], Jaesung Seo[1,7], Hyo-Kyoung Choi[2,7], Hye-Jeong Oh[1], Garam Guk[1], Yoo-Hyun Lee[3], Jeongmin Lee[4], Woo Jin Jun[5], Kyung-Chul Choi[6,*] and Ho-Geun Yoon[1,*]

[1]Department of Biochemistry and Molecular Biology, Brain Korea 21 PLUS Project for Medical Sciences, Yonsei University College of Medicine, Seoul, South Korea

[2]Division of Nutrition and Metabolism Research Group, Korea Food Research Institute, Gyeonggi-do, South Korea

[3]Department of Food and Nutrition, The University of Suwon, Kyunggi-do 445-743, South Korea

[4]Department of Medical Nutrition, Kyung Hee University, Kyunggi-do, 446-701, South Korea

[5]Department of Food and Nutrition, Chonnam National University, Gwangju, South Korea

[6]Department of Biomedical Sciences, and Department of Pharmacology, University of Ulsan College of Medicine, Seoul, South Korea

[7]These authors contributed equally to this work.

*Corresponding author. *E-mail:* choikc75@amc.seoul.kr (KCC) or yhgeun@yuhs.ac (HGY)

Fig. 1a

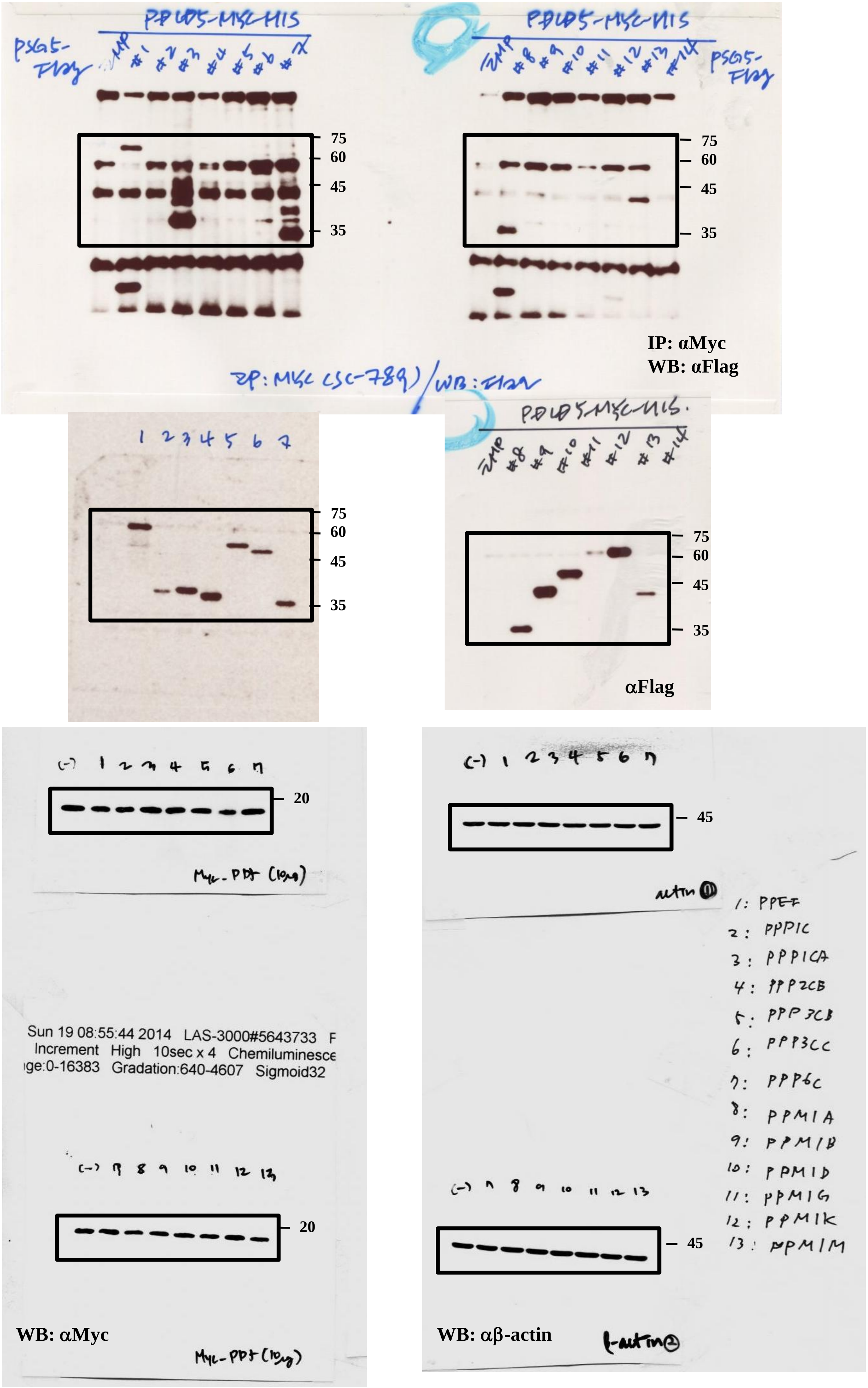
75
60
45
35
IP: αMyc
WB: αFlag
1 2 3 4 5 6 7
αFlag
(-) 1 2 3 4 5 6 7
20
45
Sun 19 08:55:44 2014 LAS-3000#5643733 F
Increment High 10sec x 4 Chemiluminesce
ge:0-16383 Gradation:640-4607 Sigmoid32
(-) 7 8 9 10 11 12 13
1: PPEF
2: PPP1C
3: PPP1CA
4: PPP2CB
5: PPP3CB
6: PPP3CC
7: PPP6C
8: PPM1A
9: PPM1B
10: PPM1D
11: PPM1G
12: PPM1K
13: PPM1M
WB: αMyc
WB: αβ-actin

Fig. 1b

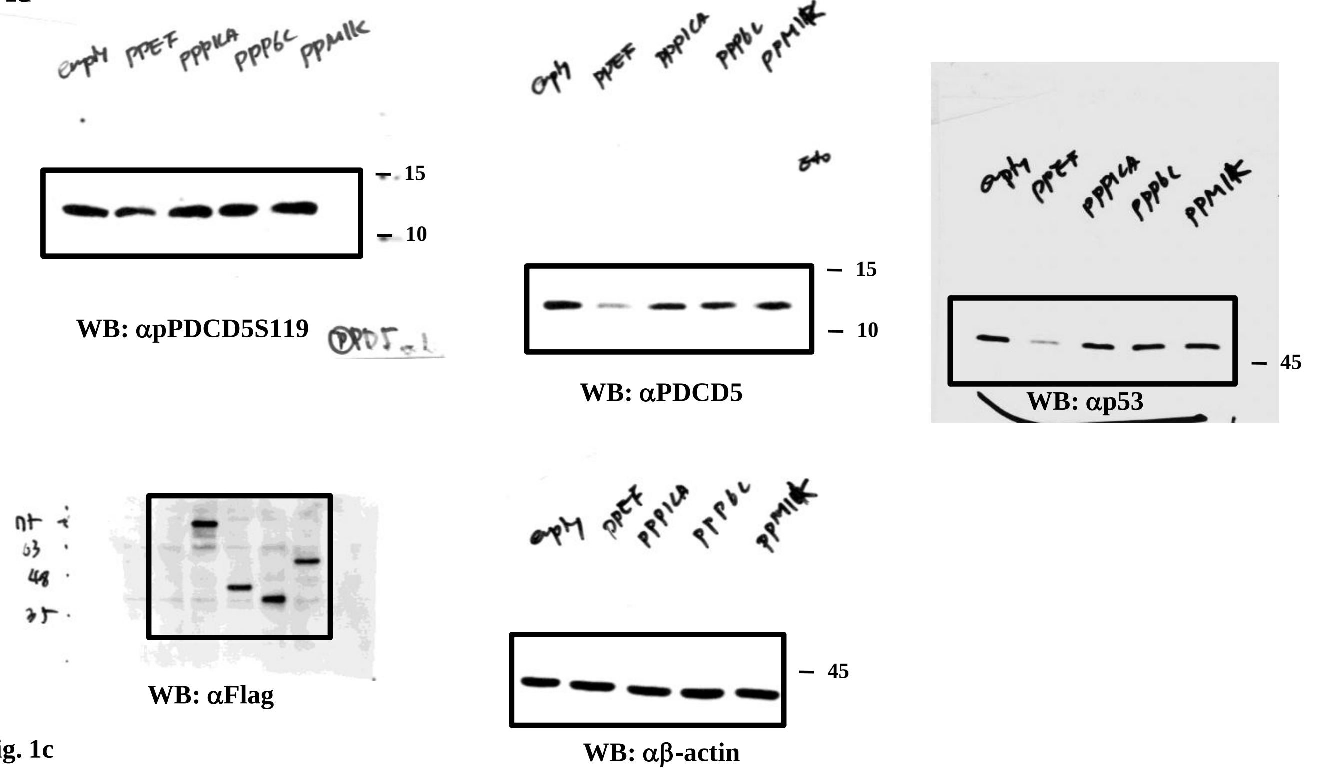

Fig. 1c

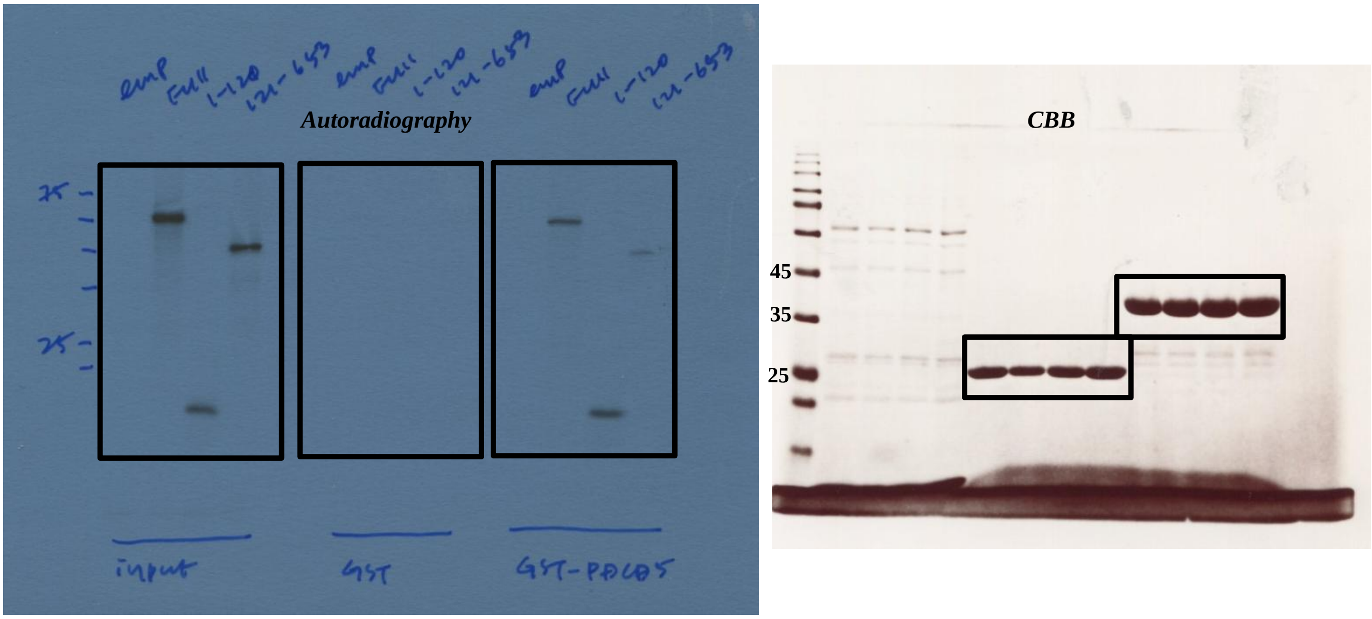

Fig. 1d

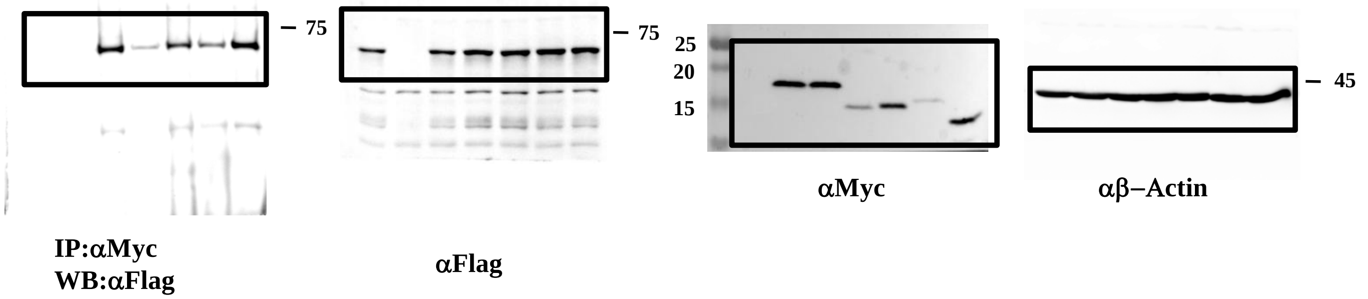

Fig. 2a

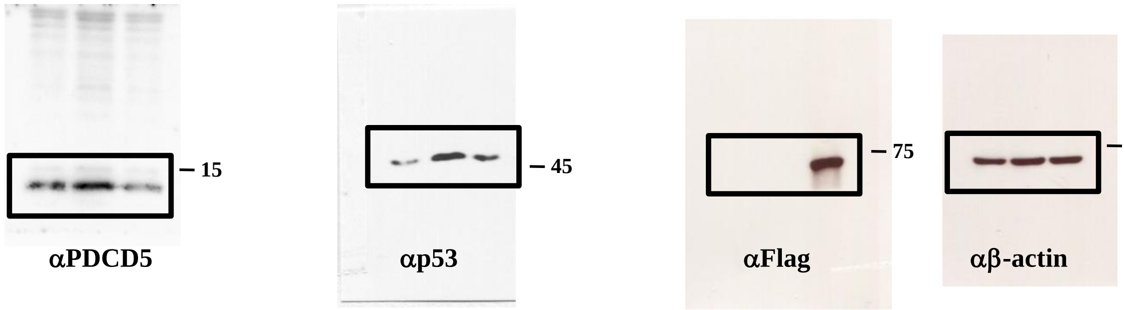

Fig. 2b

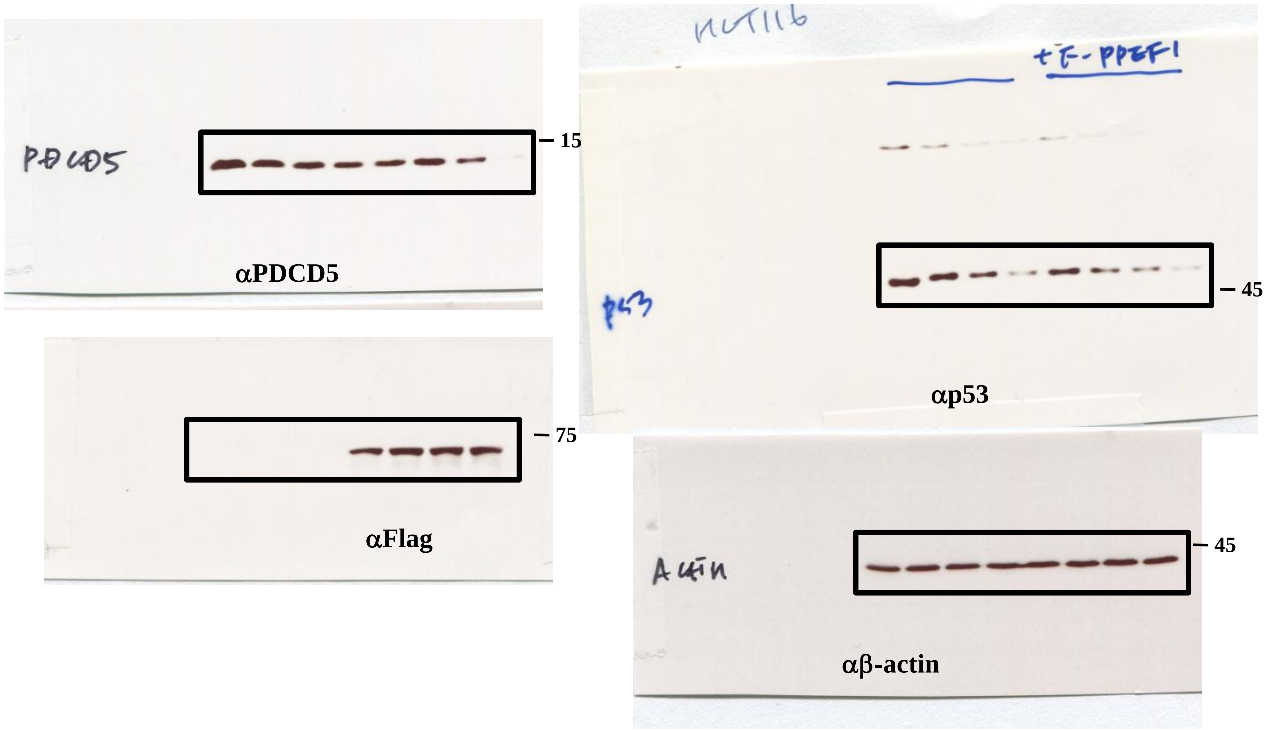

Fig. 2c

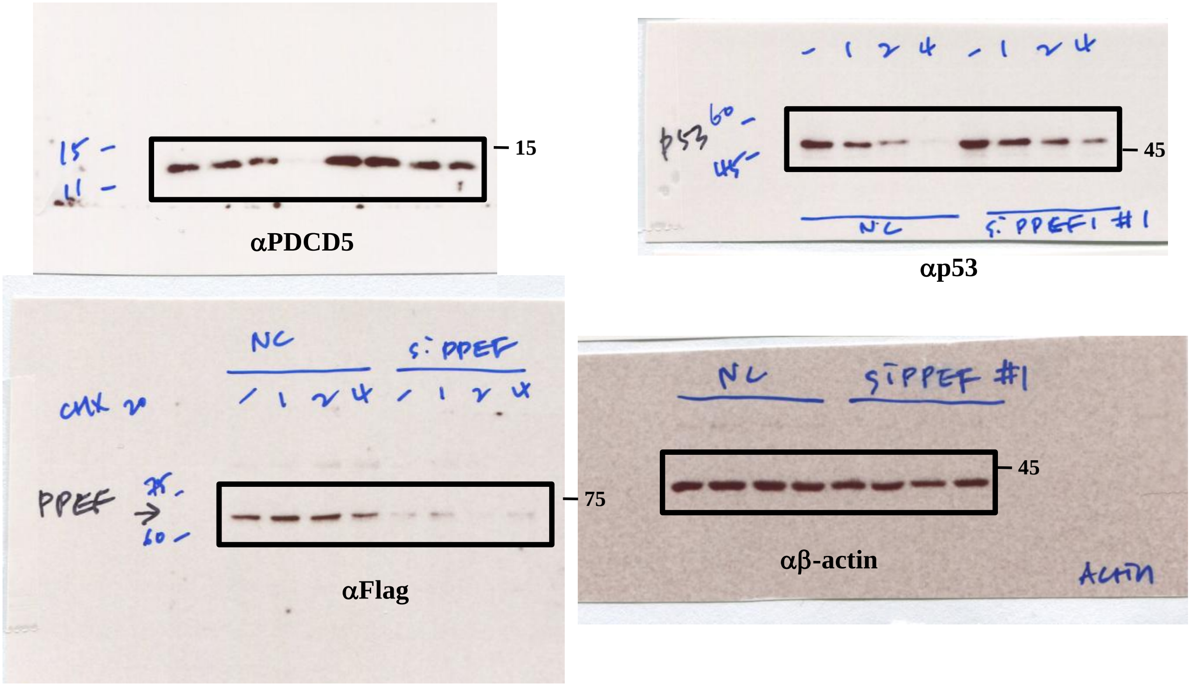

Fig. 2e

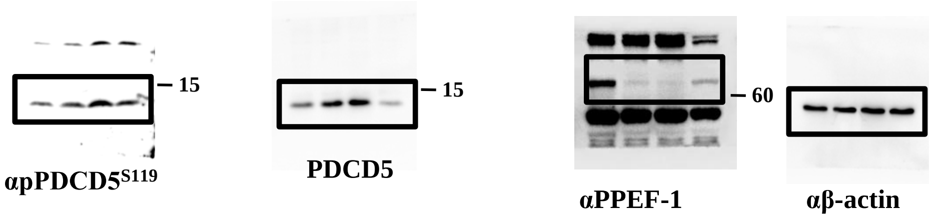

Fig. 2f

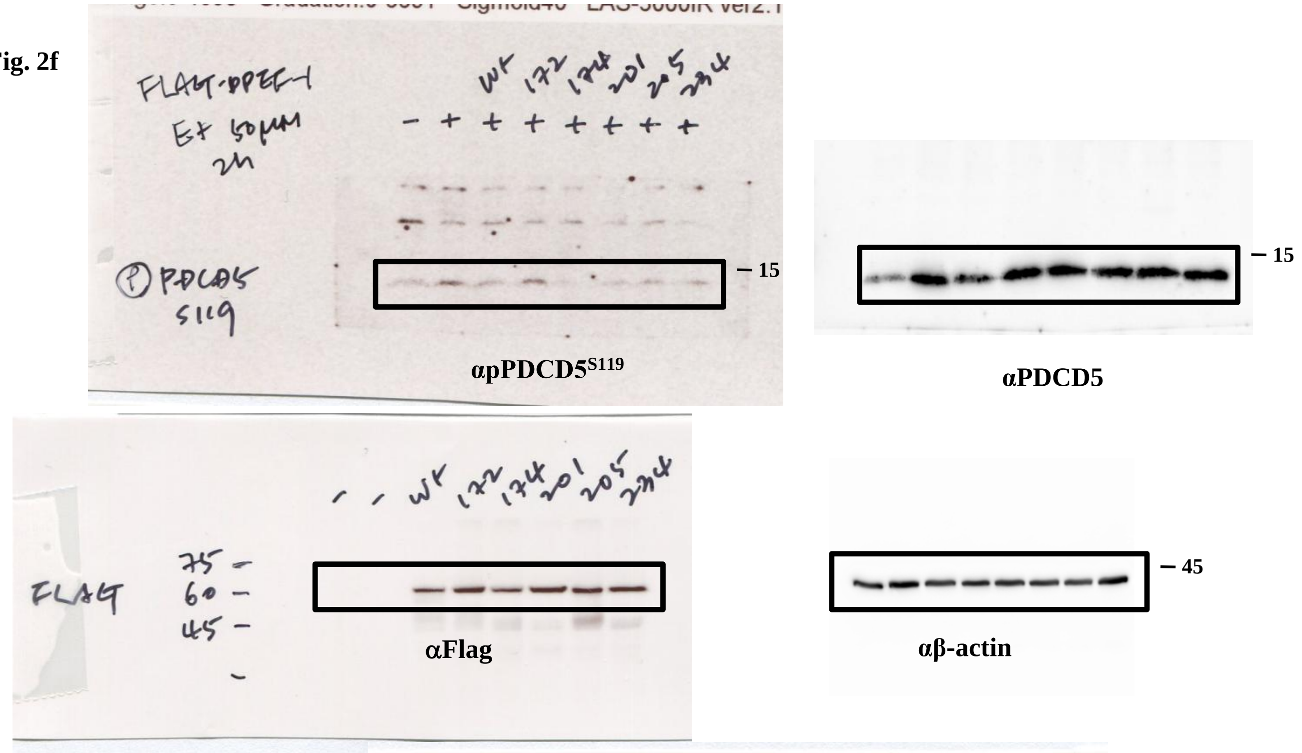

Fig. 2g

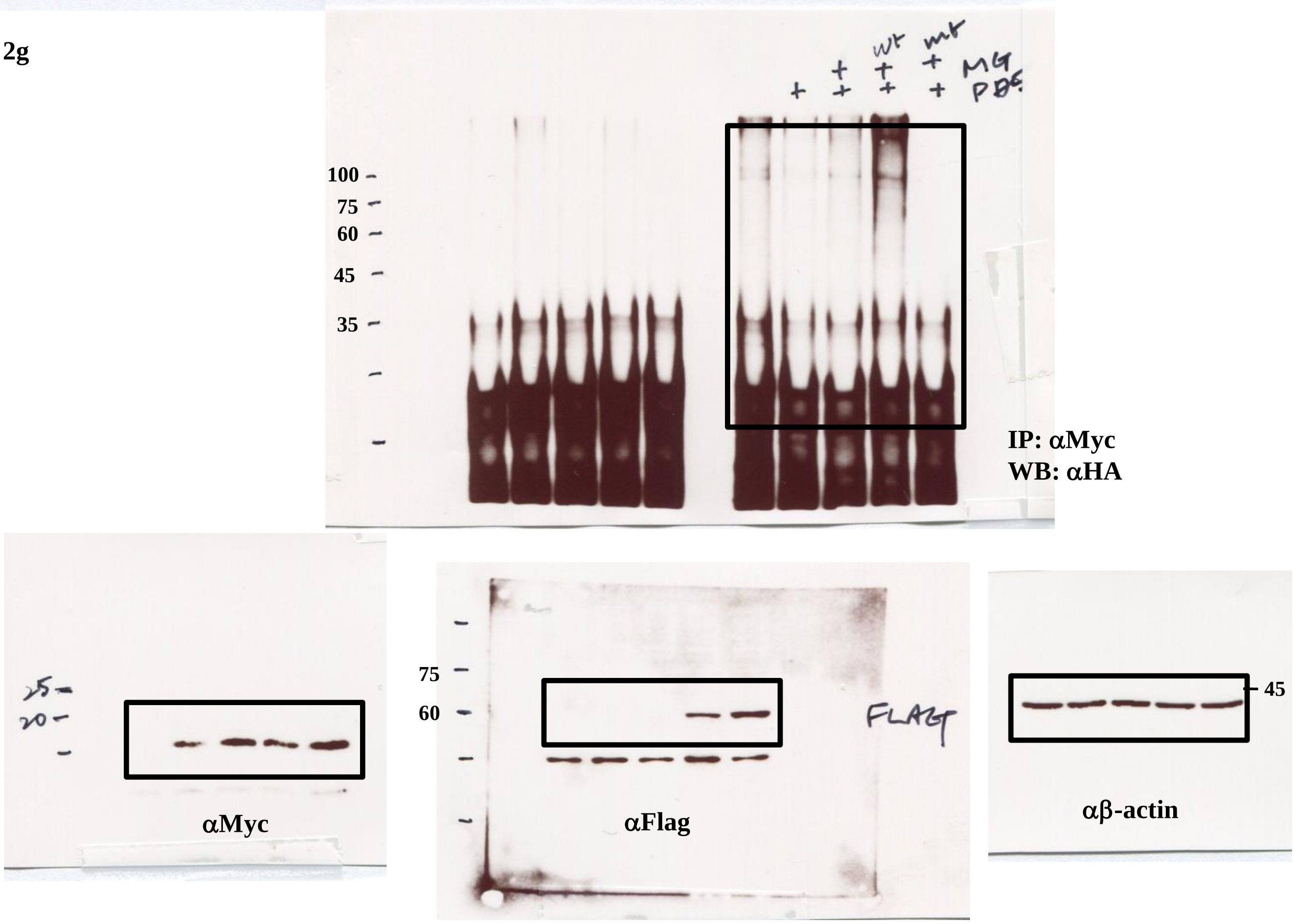

Fig. 3b

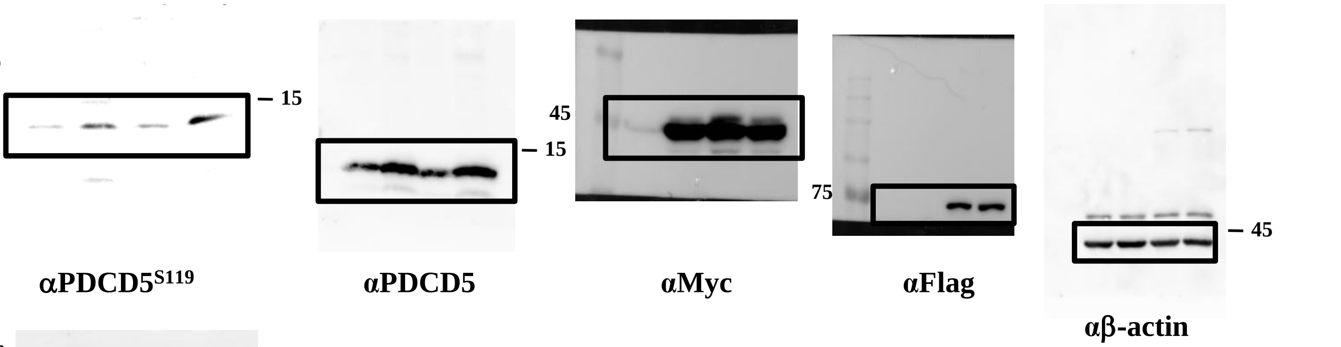

Fig. 3c

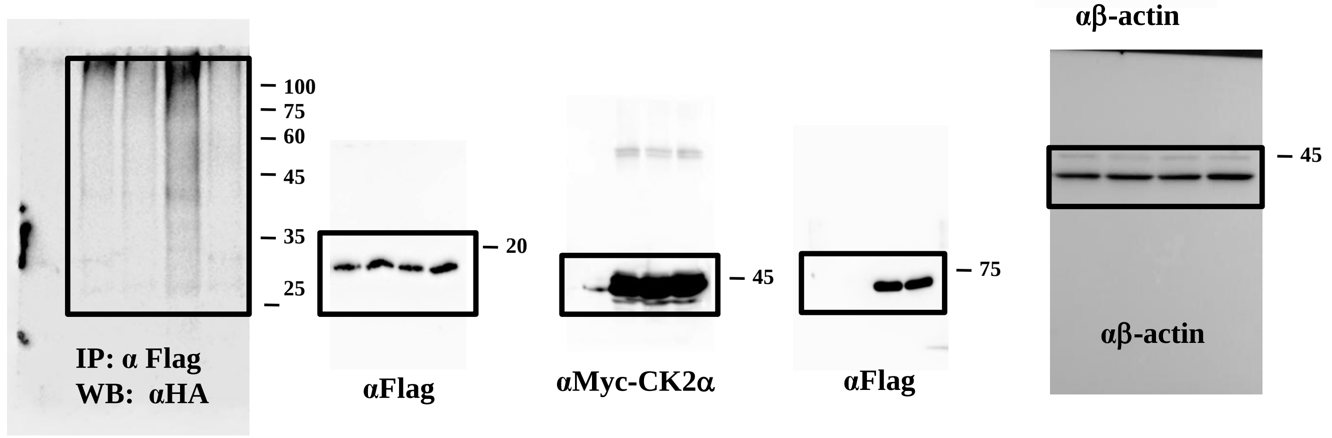

Fig. 4a

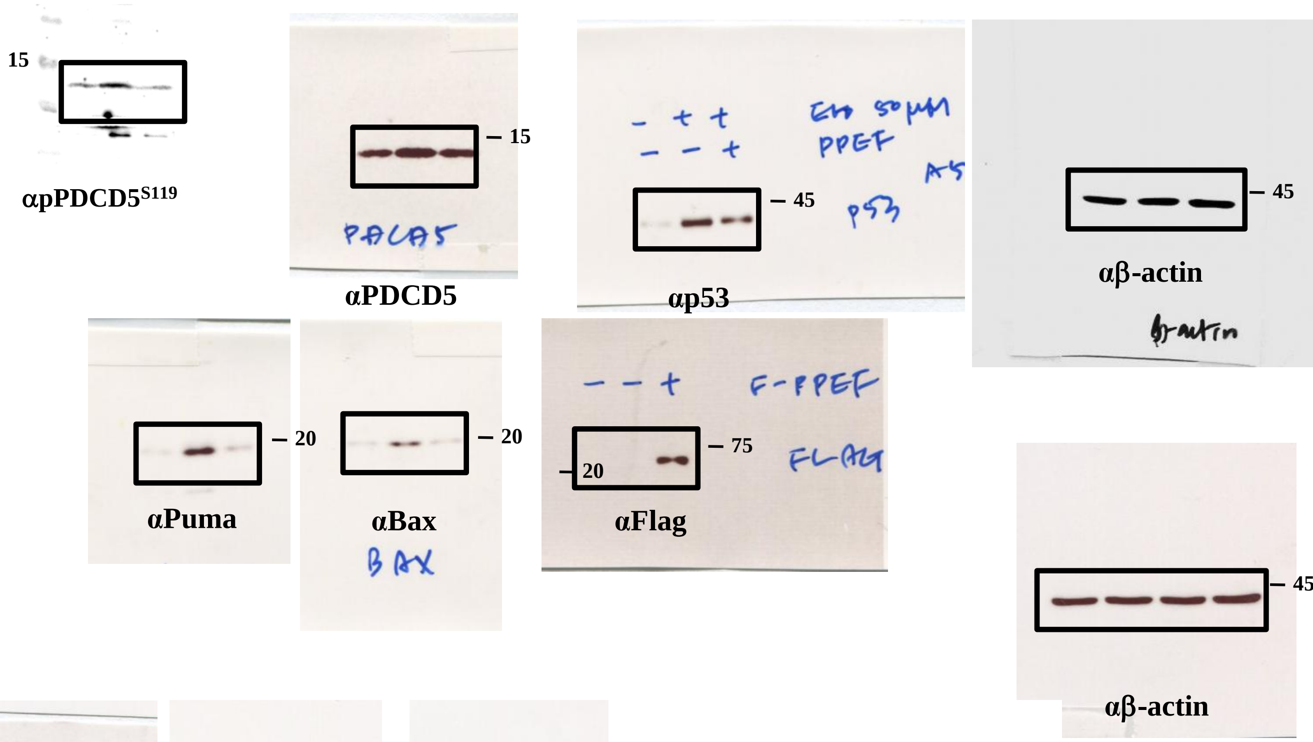

Fig. 4c

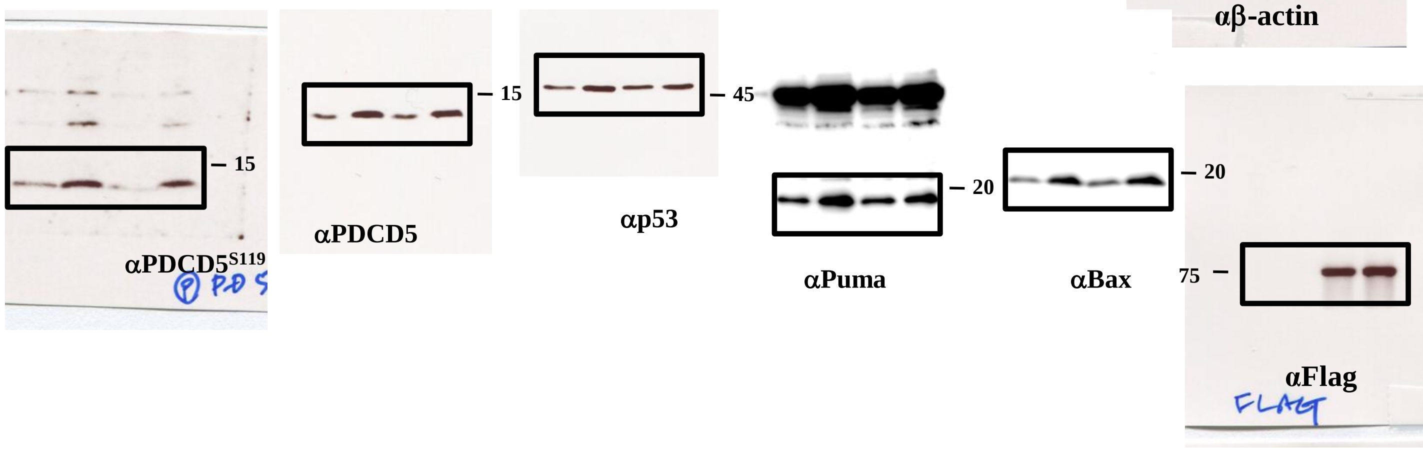

Fig. 4d

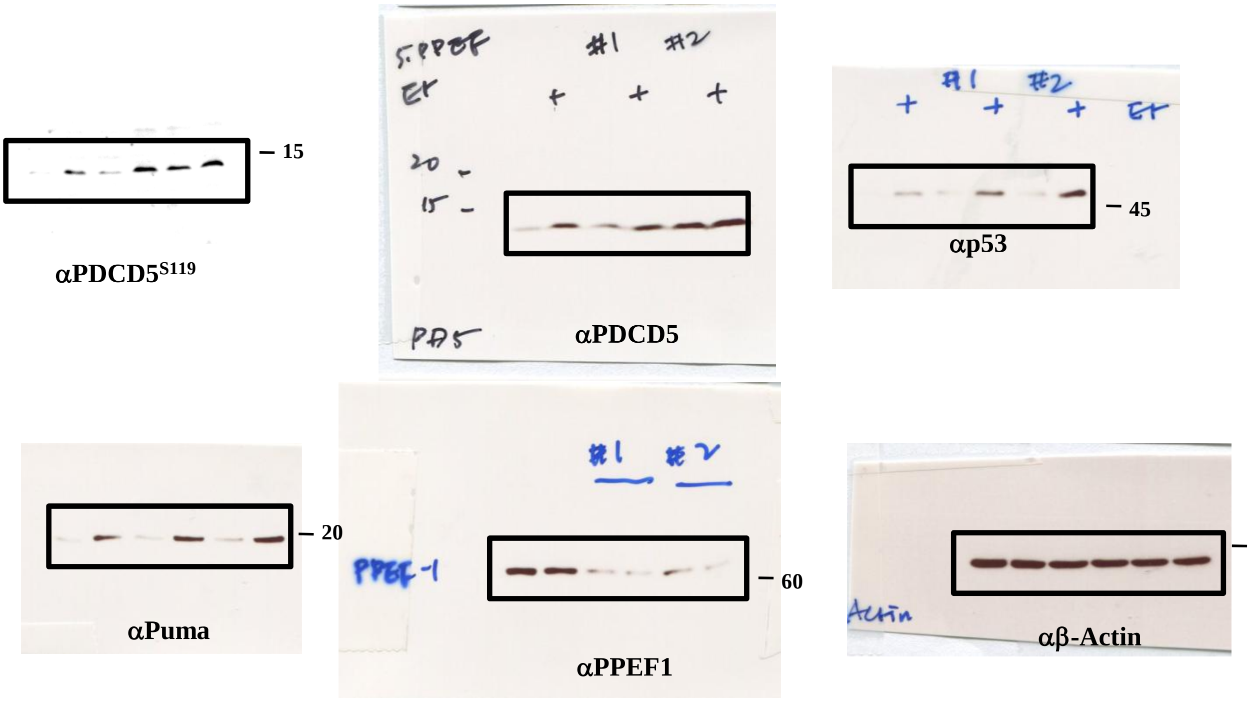

Fig. 4e

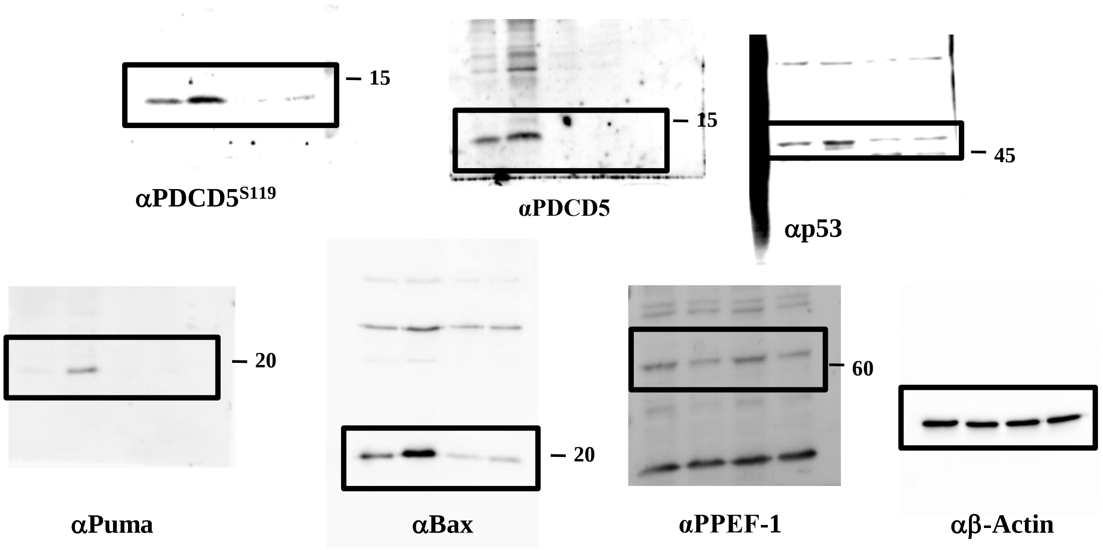

Fig. 5c

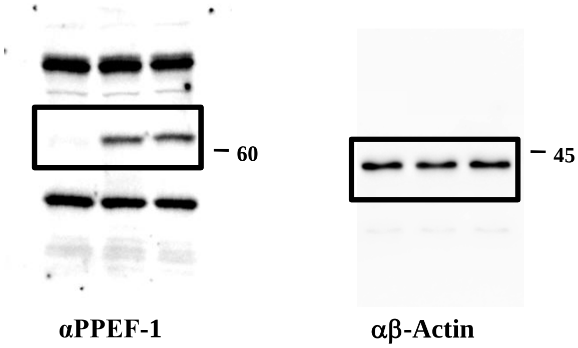

Supplement: Supplementary Information [file srep39222-s1.pdf]
